# Practical Notes on Diagnosis and Treatment

**Published:** 1906-12-01

**Authors:** 


### Practical Notes on Diagnosis and Treatment.

**Fissured Nipples.**

The following is a useful local application:—Olive oil ʒss. ichthyol, lanoline, and glycerine, of each ʒij.

**Albuminuria in Pregnancy.**

The frequent examination of the urine in pregnant women, especially in primiparæ, with a view to the early detection of albuminuria, is a course strongly to be advised.

**Snuff for Coryza.**

Salol 1 part, acid salicylic 20 parts, tannic acid 10 parts, powdered boric acid 4 parts. A pinch to be introduced into each nostril at the commencement of the symptoms, and to be repeated every hour for eight hours, but not longer.

**Flatulence.**

The following powder is recommended to relieve flatulence:—Naphthol, magnesium carbonate, and charcoal, of each 1 dram; oil of peppermint 2 minims. Divide into 15 powders, and take one at the beginning of each meal.

**Draught for Angina Pectoris.**

Patients who suffer from angina pectoris should always carry with them such a restorative draught as the following:—Sal volatile ʒj, sodium bicarbonate grs. x., comp. tinct. cardamoms ʒj., spirit of chloroform ♏xx., solution of nitroglycerine ♏ij., water to ℥iss. This should be taken on the commencement of the symptoms, and it is better to sip it slowly rather than to take it at a single gulp.—*Sir Douglas Powell.*

**Pruritus Ani.**

This condition sometimes depends on the use of coffee, and ceases when this beverage is discontinued.

**Hæmothorax.**

In a case of hæmothorax there are only two explanations worth entertaining—the one aneurism, the other malignant disease of the lung or pleura, or of both.—*Sir Wm. Gairdner.*

**Nocturnal Enuresis.**

Occasionally cases are met with in which all treatment has failed. Yet it will be found that often the condition disappears when puberty is reached, and this should be remembered in framing a prognosis.—*Mr. Pearce Gould.*

**Drug Treatment of Tubercular Testis.**

In these cases I always order grey powder in doses of $1\frac{1}{2}$ grains twice daily, and my experience is that the condition usually benefits by such treatment if persevered with sufficiently long.—*Mr. Reginald Harrison.*

**Paracentesis in Serous Pleural Effusions.**

A great deal too much stress is laid upon the question of early tapping in cases of serous pleural effusions. As a matter of fact, fluid in considerable quantities may remain in the chest for several weeks, and yet when it is removed the lung will recover perfectly. My rule is to tap only when (1) the effusion is very considerable in amount, (2) when the viscera are displaced by it, and (3) when symptoms of urgency arise.—*Dr. Goodhart.*

